# Using normalisation process theory (NPT) to explore implementation of the maternal perinatal death surveillance and response (MPDSR) policy in Uganda: a reflection

**DOI:** 10.1186/s12961-024-01191-x

**Published:** 2024-11-04

**Authors:** David Roger Walugembe, Katrina Plamondon, Frank Kaharuza, Peter Waiswa, Lloy Wylie, Nadine Wathen, Anita Kothari

**Affiliations:** 1 Faculty of Health and Social Development, School of Nursing, The University of British Columbia, Okanagan Campus, Kelowna, BC V1V 1V7 Canada; 2 Makerere University School of Public Health, P.O. Box 7072, Kampala, Uganda; 3 Department of Psychiatry, Pathology and Health Sciences, Schulich Interfaculty Program in Public Health, Western University, 1465 Richmond St., London, ON N6G 2M1 Canada; 4 Arthur Labatt Family School of Nursing, FIMS & Nursing Building, Western University, London, ON N6A 5B9 Canada; 5 School of Health Studies, Arthur and Sonia Labatt Health Sciences Building, Western University, London, ON N6A 5B9 Canada

**Keywords:** Normalisation process theory, Implementation, Maternal health, Perinatal health, Mortality, Surveillance, Response, Health policy, Uganda, Health system

## Abstract

**Background:**

The implementation of the maternal perinatal death surveillance and response (MPDSR) policy is among the envisaged strategies to reduce the high global burden of maternal and perinatal mortality and morbidity. However, implementation of this policy across various contexts is inconsistent. Theoretically informed approaches to process evaluation can support assessment the implementation of policy interventions such as MPDSR, particularly in understanding what the actors involved actually do. In this article, we reflect on how the normalisation process theory (NPT) was used to explore implementation of the MPDSR policy in Uganda. NPT is a sociological theory concerned with the social organisation of the work (implementation) of making practices routine elements of everyday life (embedding) and of sustaining embedded practices in their social contexts (integration).

**Methods:**

This qualitative multiple case study conducted across eight districts in Uganda and among 10 health facilities (cases) representing four out of the seven levels of the Uganda health care system. NPT was utilised in several ways including informing the study design, structuring the data collection tools (semi-structured interview guides), providing an organising framework for analysis, interpreting and reporting of study findings as well as making recommendations. Study participants were purposely selected to reflect the range of actors involved in the policy implementation process. This included direct care providers located at each of the cases, the Ministry of Health and from agencies and professional associations. Data were collected using semi-structured, in-depth interviews and were inductively and deductively analysed using NPT constructs and subconstructs.

**Results and conclusion:**

NPT served useful for process evaluation, particularly in identifying factors that contribute to variations in policy implementation. Considering the NPT focus on the agency of people involved in implementation, additional efforts are required to understand how recipients of the policy intervention influence how the intervention becomes embedded within the various contexts.

## Contributions to the literature

This paper contributes towards addressing the gap of limited evidence on the use of implementation science theoretical approaches in studying and assessing the implementation of health policy interventions among low-income contexts.

Offers practical insights on how implementation science theoretical approaches such as NPT can be used to explore implementation of health policy interventions in low-income contexts.

Demonstrates the benefits and limitations of using theoretical approaches to study and assess the implementation of health policy interventions to improve maternal and child health in low-income contexts.

## Background

A maternal death is defined as the death of a woman while pregnant or within 42 days of termination of pregnancy, irrespective of the duration and site of the pregnancy, from any cause related to or aggravated by the pregnancy or its management but not from accidental or incidental causes; perinatal mortality refers to the number of stillbirths and deaths in the first week of life [94]. The global burden of maternal and perinatal mortality and morbidity remains high with an estimated 289,000 maternal deaths, 2.6 million stillbirths and 2.4 million newborn deaths [5, 85, 87, 96]. An estimated 75% of these deaths occur in low- and middle-income countries (LMICs) with 62% specifically occurring in sub-Saharan Africa [13, 48, 92]. The most common causes of maternal deaths include: haemorrhage, hypertensive disorders and pregnancy related sepsis; for neonatal deaths, the most common causes include birth asphyxia, newborn sepsis and prematurity [35, 42, 79].

Efforts to counter the high burden of maternal and perinatal mortality and morbidity were prioritised within the Millennium Development Goals (MDGs) and their targets, specifically: (1) MDG 4 – improvement of child mortality through under-five mortality reduction by two thirds and (2) MDG 5 – the improvement of maternal health by reducing the maternal mortality ratio by 75% between 1990 and 2015 [86]. While some countries (Bolivia, Bhutan, China, Egypt, Equatorial Guinea, Eritrea and Rwanda) made substantial progress towards the attainment of MDGs 4 and 5, many others, especially in sub-Saharan African region, have made insufficient progress or none at all [41, 96]. It is against this background that renewed efforts under the Sustainable Development Goals (SDGs) seek to reduce the global maternal mortality ratio to less than 70 maternal deaths per 100,000 live births and reduce newborn mortality to at least as low as 12 per 1000 live births in every country by 2030 [14].

Among the strategies to reduce maternal and perinatal deaths is the implementation of maternal perinatal death reviews or audits. A maternal/perinatal death audit/review is an in-depth systematic review of maternal/perinatal deaths to delineate their underlying health, social and other contributory factors and the lessons learned from such an audit are used in making recommendations to prevent similar future deaths [58, 61]. WHO initiated the MPDRs to go beyond the numbers captured by measures such as maternal mortality ratio (MMR) and infant mortality rate (IMR) and facilitate understanding of the underlying reasons why women and their newborns die as well as devise contextually appropriate remedial actions [44, 93]. The WHO handbook, “Beyond the numbers: reviewing maternal deaths and disabilities to make pregnancy safer” [93], describes several strategies for reviewing cases of maternal deaths and disabilities to help understand why mothers and their newborns die. These approaches include community-based (verbal autopsy), facility-based maternal death reviews, confidential enquiries into maternal deaths, surveys of severe morbidity and clinical audits, among others. Each of these approaches can be implemented at the community, healthcare facility, regional or national level. Authors have noted that community, facility-based reviews and confidential enquiries into maternal deaths are among the easiest to introduce, promote and implement in resource-constrained contexts [44, 93]. The overarching purpose of each of these approaches is to provide lessons and act on the recommendations of the reviews.

As observed by Kinney et al. [37], the implementation of these strategies has evolved from clinical obstetric to maternal death reviews (MDRs) and/or perinatal death reviews (PDRs), maternal death surveillance and response (MDSR) and currently maternal perinatal death surveillance and response (MPDSR) [37]. Prior to 2012, much of the focus was on MDRs and/or PDRs. However, according to Smith et al. [81], in 2012, the WHO and partners introduced the maternal death surveillance and response (MDSR) as a new approach aimed at collecting and using robust information for decision making [81]. Kinney et al. [37] further observe that the integration of the perinatal death element into MDSR was first reported in 2016.

The evolution of these strategies has led to a high degree of variability in how audits are understood and implemented across various contexts within and across health systems. For example, according to WHO [96], 34 out of the 71 high priority countries responding to the WHO Global Maternal, Newborn Child and Adolescent Health (MNCAH) 2013–2014 policy survey had policy on notification of all maternal deaths to a central authority within 24 h of the event [96]. Additionally, 53 countries had a policy requiring all maternal deaths to be reviewed. Policy support to perform health facility-based maternal death reviews was reported in 55 countries, while the processes to perform community maternal death reviews were in place in 30 countries. Routine reviews at facility and community levels were reportedly confined to the subnational or subregional level and are not part of the essential MDSR cycle where national level surveillance and response are key components [96]. Furthermore, only 20 out of the 71 high priority countries had a national panel/committee in place to review maternal deaths every quarter each year [48, 96].

However, according to WHO [96], there is a renewed interest among LMICs in having a national notification policy for all maternal deaths. As such, the number of countries with such a policy is reported to have increased from 51 in 2009 to 93 by 2016 [97]. In addition, a new WHO document titled “Maternal and Perinatal Death Surveillance and Response (MPDSR): Materials to Support Implementation” was recently launched with an aim of providing a roadmap for conducting MPDSR in clinical and policy settings [98].

## Aim of the article

The aim of this article is to reflect upon how the NPT was used to explore implementation of the MPDSR Policy in Uganda. It arose from a larger study that explored variations in the determinants of implementation of a health systems level policy intervention to improve maternal and child health [91].

Uganda is one of the 71 high priority and 110 low- and middle-income countries that responded to the WHO global MNCAH policy indicator survey and the WHO-UNFPA MDSR baseline surveys [96]. However, there exist reporting discrepancies regarding the implementation of MPDSR in Uganda. For example, according to the WHO Global MDSR Implementation Survey 2015, Uganda is among 89% of the countries that reported having a national maternal death notification policy and 88% of the countries that had a policy to review such deaths. Additionally, the implementation of the maternal and perinatal death reviews/MPDSR was reported to be taking place both at national and subnational levels with the involvement of civil society at national level and community representation at the subnational death review committee. Although the country profile indicates that there were no data regarding existence of a subnational MPDSR committee, Uganda was among 76% of the countries with a national MPDSR committee and 67% of the countries with a subnational MPDSR committee [67]. Furthermore, although 48% of the countries that had a national committee reported meeting at least biannually as recommended by the MPDSR guidelines, data on Uganda’s country profile indicate that the national committee meets on a quarterly basis every year. Whereas these policies were reportedly adopted in 2009 and 2011, respectively [97], publicly available reports indicate that the Ugandan Ministry of Health mandated health facilities to report maternal and perinatal deaths and to audit maternal and perinatal death reviews in 2008 [66].

Despite reducing from 435 maternal deaths per 100,000 live births in 2006 to 310/100,000 live births in 2010 [95] and from 70 perinatal deaths per 1000 total births to 38/1000 total births [70], Uganda’s maternal and perinatal mortality rates still remain unacceptably high. Additionally, previous reports observed that as of 2011, 87% of maternal and perinatal deaths were not being reported to the Ugandan Ministry of Health by the health units [66] and that only a handful of health facilities had been trained on the implementation of the MPDR policy [63]. Furthermore, there was an observed limited emphasis on perinatal death reviews [67]. These observed discrepancies in data necessitated further exploration of implementation of MPDSR policy in Uganda to understand what explains the variations in the determinants of implementation of this policy in the various settings and what the stakeholders involved actually do to implement it.

Effectively studying and assessing the implementation of policy interventions such as MPDSR among LMICs as well as understanding what the actors involved actually do, however, can benefit from theoretically informed approaches. Theoretical approaches provide a better understanding and explanation of how and why implementation succeeds or fails [26, 37, 57, 71]. Additionally, the use of theory to study the implementation of interventions offers generalisable frameworks that can apply across differing settings and individuals, and offers the opportunity for incremental accumulation of knowledge as well as explicit frameworks for analysis [17, 20, 26, 29, 60]. Helfrich et al. [29] observe that using theory not only enhances understanding of barriers to implementation but may enhance the ability to design and improve implementation processes [29].

Understanding the implementation of policy interventions such as MPDSR requires understanding of both the processes involved and how the intervention becomes workable and integrated into everyday work [55]. Thorsen et al. [85] observe that studies on the implementation of MPDSR have focused on the entirety of the MPDSR process with heavy emphasis on establishing a committee and implementing the recommendations as a way to institutionalise them. However, to understand the variations in the implementation and integration of maternal and perinatal death reviews, there is a need to look at what people actually do and how they work. As noted by May and Finch [49], embedding of a practice is dependent on organised and organising agency [49] and requires continuous investment in sense-making, commitment, effort and appraisal of the routinisation of a complex intervention [54].

Despite the benefits of using theoretically informed approaches to study implementation of maternal and child health policy interventions, only a few studies have explicitly articulated their theoretical underpinnings. These include the stages of change model [9] and the strength, weakness opportunities and threats (SWOT) analysis framework [40], which were used to analyse data and describe study findings. The limited use of theoretically informed approaches to study and assess the implementation of maternal and child health policy interventions such as MPDSR among LMICs may account for our limited understanding of their implementation as well as their reported minimal impact in reducing maternal and perinatal mortality and morbidity [16, 26, 37, 39, 59, 77]. As such, theoretically informed efforts may be helpful to explain the causes in variations in the implementation of the interventions within and across health systems [25, 37, 46, 75].

### Implementation theories, models and frameworks

With advances in implementation science, numerous theories, models and frameworks have been developed or adapted for potential use in addressing various implementation challenges [26, 71]. Several reasons have been advanced to explain the increasing interest and focus on the use of theories, models and frameworks. First is the increasing recognition that a poor theoretical underpinning makes it challenging to understand and explain how and why implementation succeeds or fails, which subsequently hinders the development of strategies to achieve more successful implementation [16, 17, 71]. Second is the desire to gain more insights into the mechanisms by which implementation is more likely to succeed or not [16, 26, 29, 71]. Nilsen [71] observes that in implementation science, theories, models and frameworks have three overarching aims: describing and/or guiding the process of translating research into practice, understanding and or explaining what influences implementation outcomes and evaluating implementation [71]. Against this background, he provided a taxonomy of five categories of theories, models and frameworks used in implementation science and these include process models, determinant frameworks, classic theories, implementation theories and evaluation frameworks. Nilsen, however, notes that these categories are not always recognised as separate types of approaches in literature [71]. Following a review of these theories, models and frameworks, this study opted to use the NPT to explore the research questions.

The rationale for selecting this theory was informed by the fact that NPT combines the merits of multiple theoretical approaches and disciplines from which it was drawn and was envisaged to offer a more complete understanding and explanation of certain aspects of implementation [72]. Additionally, its intent, level of abstraction, evidence of utilisation in previous empirical studies, provision of how-to support tools and dual purpose as a theory and evaluation framework, made NPT a suitable option for pursuing the study objectives and research questions. Above all, the four NPT constructs and their respective subdomains as described in detail below were considered sufficient to support the exploration of the study objectives [49, 50, 52, 54, 57].

### About the normalisation process theory-evolution of NPT

Developed between 2000 and 2009, NPT is a sociological theory concerned with the social organisation of the work (implementation) of making practices routine elements of everyday life (embedding) and of sustaining embedded practices in their social contexts (integration) [49, 53, 54, 57]. It seeks to provide a set of sociological tools that facilitate understanding and explanation of the social processes through which new or modified practices of thinking, enacting and organising work are operationalised in healthcare and other institutional settings [49, 53, 54]. Within the context of this theory, normalisation refers to work that actors do as they engage with some ensemble of activities (that may include new or changed ways of thinking, acting and organising) and by which means it becomes routinely embedded in the matrices of already existing, socially patterned, knowledge and practices [49, 53, 54].

### NPT constructs

**Fig. 1:**
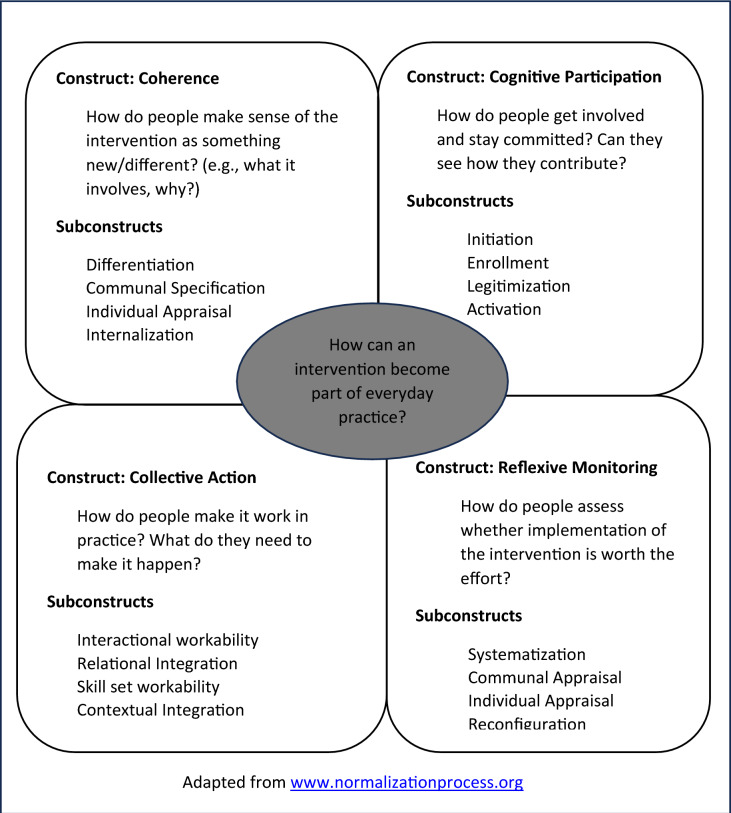
NPT constructs

As illustrated in Fig. 1, NPT has four constructs – coherence, cognitive participation, collective action and reflexive monitoring – that were used to address the proposed study research questions [49, 53, 54]. Each of these constructs has four subcomponents that further explicate what the construct is about and how it can facilitate exploration of the implementation of an intervention within its social contexts. A confirmatory factor analysis [28] of the items supported the NPT proposition that embedding of a new practice requires that participants be involved in the process to engage in work across the four constructs [18, 76]. Additionally, tests of internal consistency supported the use of the items either as an overall measure of normalisation or as four construct measures.

### Coherence

Coherence refers to how people understand and make sense of a practice. It concerns the sense-making work that people do individually and collectively when they are faced with the problem of operationalising some set of practices. Coherence is comprised of four subcomponents including differentiation, communal specification, individual specification and internalisation. According to the NPT toolkit [51], differentiation speaks to how a set of practices and their objects are different from each other while communal specification refers to how people working together build a shared understanding of the aims, objectives and benefits of a set of practices. Individual specification refers to how participants collaboratively need to do things that will help them to understand their specific tasks and responsibilities around a set of practices. Internalisation speaks to how participants in sense-making undertake efforts to understand the value, benefits and importance of a set of practices.

### Cognitive participation

Cognitive participation refers to the relational work that people do to build a community of practice around a new technology or complex intervention. It specifically focuses on how people engage and participate with a practice and entails the sub-components of initiation, enrolment, legitimating and activation [49, 51]. Initiation is concerned with who drives forward the work implementing a new or modified set of practices while enrolment looks at how such participants organise or reorganise themselves to collectively contribute to the work involved in the implementation of a practice/intervention. Legitimation, which is the third subcomponent of cognitive participation, refers to the work that goes into interpreting and buying into a practice by other actors and ensuring that that they can make a valid contribution [49]. It facilitates making collective decisions among actors on procedures by which a practice is to be enacted and how engagement with it is defined [49]. The work of decision making in legitimation leads to activation of a practice which is the fourth subcomponent of cognitive participation. It refers to how participants collectively define the actions and procedures needed to sustain a practice and how they can stay involved [51].

### Collective action

Collective action refers to the operational work that people do to enact a set of practices [49, 51]. The four subcomponents of collective action are: interactional workability (how actors operationalise a practice), relational integration (the way a practice is mediated and understood within the networks of people around it), skill set workability (the distribution and conduct of work that distributes a practice in division of labour) and contextual integration (incorporation of a practice within a social context) [49, 54].

### Reflexive monitoring

Reflexive monitoring as the fourth construct of NPT refers to the appraisal work that people do to assess and understand the ways that a new set of practices affect them and others around them [49, 51]. Just like the constructs discussed above, reflexive monitoring has four sub-components. These include: systematisation, communal appraisal, individual appraisal and reconfiguration [49, 51]. Systematization refers to the work undertaken by participants/actors to determine the effectiveness and usefulness of new set of practices to them and others involved [49, 51]. Communal appraisal is described as the work that formal and informal groups do to evaluate the worth of a set of practices [49, 51]. Communal appraisals draw on a variety of experiential and systematised information to accomplish their work. Individual appraisal refers to the work done by participants in a new set of practices to appraise its effects on them and the contexts in which they are set [49, 51]. It relies on experiential and unsystematic practices of judging the value and outcomes of an intervention [49]. It is from this work that actions through which individuals express their personal relationship to a complex intervention emerge. As a result of both communal and individual appraisal, reconfiguration, which refers to the attempts to redefine procedures or modify practices and even to change the shape of the intervention itself, emerges. Reconfiguration facilitates provision of feedback into the constructs of coherence and the meaningfulness of a practice [49, 51].

### Utility of NPT

As a middle-range theory [54], NPT goes beyond the description of barriers and facilitators to understanding how the things that people do when they implement interventions such as the MPDSR policy become routinely embedded in their social contexts [49]. Two systematic reviews by McEvoy et al. [57] and May et al. [52] explored how NPT has been used in studies of implementation processes including feasibility studies and process evaluations of complex healthcare interventions [52, 57]. McEvoy et al. [57] observed that in almost all the 29 studies included in their systematic review, NPT was used as an organising framework for analyses, reporting of findings and to inform study intervention design [24, 34, 45, 57]. NPT was also used to generate research questions for fieldwork [27, 32, 78] and create tools for investigating and supporting implementation [19, 53, 57]. True to its original intentions, most NPT studies included in the systematic review were from the field of e-health and telehealthcare (21 studies), while others explored various healthcare fields such as chronic health care, maternity care and language interpretation services [57]. Various authors who provided their experiences of using NPT (20/29) acknowledged the benefits of utilising the theory. For example, 15 out of the 20 authors acknowledged that it was beneficial and provided an explanatory framework for helping to identify factors that promote and or inhibit implementation of complex interventions [21, 23, 47, 53]. Others lauded NPT for assisting them to make clear recommendations for future implementation [6, 57], and some acknowledged the positive impact the theory had on the trial design and intervention development [21, 24, 56].

Similarly, May et al. [52] noted that NPT has been used to provide researchers and practitioners with a conceptual vocabulary for rigorous studies of implementation processes [52]. NPT was reported to help identify, characterise and explain empirically identifiable mechanisms that motivate and shape implementation processes. Furthermore, it was noted that analyses using NPT can effectively assist in the explanation of the success or failure of specific implementation projects [52]. May et al. [52] identified 108 studies of complex healthcare interventions and related implementation processes reported in 130 journal articles and published after 2008. Specifically, NPT was employed in 26 controlled and 82 uncontrolled studies ranging from complex intervention trials, intervention design studies, feasibility studies, process evaluations of field studies, among others. The majority of these studies focused on service organisation and delivery (29), followed by diagnostic and therapeutic interventions (28), e-health and telemedicine (21), implementation of screening and surveillance tools (11), decision support and shared decision making (8) and implementing change in professional roles (7), as well as guideline implementation (4). These studies were predominantly retrospective in nature and used qualitative methods (72), albeit a few employed mixed methods (7), surveys (2) and one prospective cohort study. Among the noted benefits of using NPT was the ability to depict elements of the implementation processes and how the constructs of the theory could be applied in a stable and consistent way within and between studies [52]. Additionally, the provision of conceptual tools for a large body of feasibility studies and process evaluations of complex healthcare interventions as well as explanation of the outcomes of such studies were noted as beneficial by the authors [2, 52]. Above all the flexibility and ease of comprehension by researchers and practitioners with diverse professional backgrounds working across a variety of health care settings were also reported as key considerations informing the utilisation of NPT [10, 22, 52].

### NPT limitations

Despite the noted benefits and considerations for using NPT, there are limitations associated with the theory and its utilisation in understanding and explaining the implementation of complex interventions in various healthcare settings [52, 57]. Generally, as an implementation theory, NPT was developed out of modification and adaptation of existing approaches and this might mask contrasting assumptions and key issues that may deter exhaustive understanding and explanation [49, 71]. Additionally, the different approaches from which NPT draws may require different methods and might be based on different epistemological and ontological assumptions [71]. For example, given its sociological origins, NPT is not focused on the relationship between individual attitudes, intentions and outcomes, which is the concern of psychological theories such as the theory of planned behaviour change [2, 57]. Such a limitation hinders exhaustive exploration of how attitudes influence and or affect the implementation processes. Specifically, the most common criticisms raised across reviews of studies that have used NPT include: the overlap between the constructs [1, 30, 43, 45], over emphasis on individual and collective agency at the expense of context [15, 80], challenges with the technical vocabulary which in turn complicates coding of the qualitative data [1, 30, 34, 43] and presentation of a normative model of implementation that pays insufficient attention to idealised temporal aspects of implementation [3, 4]. In addition to these criticisms, most implementation studies that reported using NPT have been conducted within and across high income contexts with a majority conducted in the United Kingdom [52, 57]. Furthermore, only a few studies have explored the implementation of interventions to improve maternal and child health, all in high income contexts [8, 31, 33, 83].

However, studies using NPT to explore implementation of complex health interventions including those related to maternal and child health in low-and middle-income settings are beginning to emerge [6, 11, 36]. For example, Khowaja et al. [36] used NPT to guide the design of a feasibility study aimed at exploring enabling and impeding factors for the implementation of the trial of community level interventions for pre-eclampsia and eclampsia in Nigeria, Pakistan and Mozambique [36]. Additionally, Bocoum et al. [11] used the NPM to identify barriers and facilitators to the introduction of on-site antenatal syphilis screening in Burkina Faso [11]. Nonetheless, these studies are still limited in number and as such, there remain gaps in understanding the utility and applicability of NPT to explore implementation of complex interventions within healthcare settings in resource constrained contexts.

In comparison with other interventions, fewer studies have reported utilising NPT to explore implementation of complex health policy related interventions at the broader health system level [52, 57]. As May et al. [52] noted, previous attempts were mainly focused at the micro level [52]. Efforts to study implementation of health system level complex interventions are being encouraged. For example, Tazzyman et al. [84] used NPT to explore the implementation of medical revalidation in the United Kingdom [84]. Additionally, studies of guideline implementation in a diverse range of fields and conditions have been documented [7, 68, 69, 73, 89]. Most of these studies, however, have mostly been conducted in high-income context further emphasising the need to use NPT to explore implementation of complex interventions within healthcare settings in resource constrained contexts.

## Methodology

The above four major NPT constructs (coherence, cognitive participation, collective action and reflexive monitoring) and their respective subconstructs were utilised in a qualitative multiple case study aimed at exploring variations in the implementation of a health systems level policy intervention to improve maternal and child health in a low-income setting. This study was conducted in Uganda, which is among the countries with a high burden of maternal mortality currently estimated at 310/100,000 live births and high perinatal mortality estimated at 70 deaths per 1000 total births [96]. The study was conducted across eight districts in Uganda and among 10 health facilities (cases) that were selected to represent four out of the seven levels of the Uganda health care system (health center III, health center IV, general hospitals and regional referral hospitals). These levels of care were mandated by the Ministry of Health to implement the MPDR/MPDSR policy effective 2008 [66].

Uganda’s National Health System (UNHS) is made up of the public and the private sectors. The public sector includes all government of Uganda health facilities under the MoH, health services of the Ministries of Defense (Army), Education, Internal Affairs (Police and Prisons) and Ministry of Local Government (MoLG). The private health delivery system consists of private-not-for-profit (PNFPs) providers, private health practitioners (PHPs) and the traditional and complementary medicine practitioners (TCMPs). The provision of health services in Uganda is decentralised with districts and health sub-districts (HSDs) playing a key role in the delivery and management of health services at those levels. The health services are structured into national referral hospitals (NRHs) and regional referral hospitals (RRHs), general hospitals (GHs), health centre (HC) IVs, HC IIIs, HC IIs and village health teams (HC Is) [64].

Data were collected between January and May 2018 and January and April 2019. Sampling occurred at two levels: districts and cases within districts. The eight districts, were purposively selected for maximum variation to facilitate learning about a range of experiences in implementation of the MPDSR policy [82]. The selection of the districts was informed by reviewing district performance trends in district league tables [38] published in the annual health sector performance reports [62]. The annual health sector performance reports also provided an account of the performance of all districts on several health-related indicators, including notifying the Ministry of Health on the number of maternal and perinatal deaths per district local government from 2003/2004 when the district league table was launched [38]. As such, a review of the annual health sector performance reports from financial years 2003/2004 to 2017/2018 when data collection for the study commenced, facilitated observation of the performance trends for the districts that were selected for inclusion in this study and from which cases were eventually selected.

**Fig. 2:**
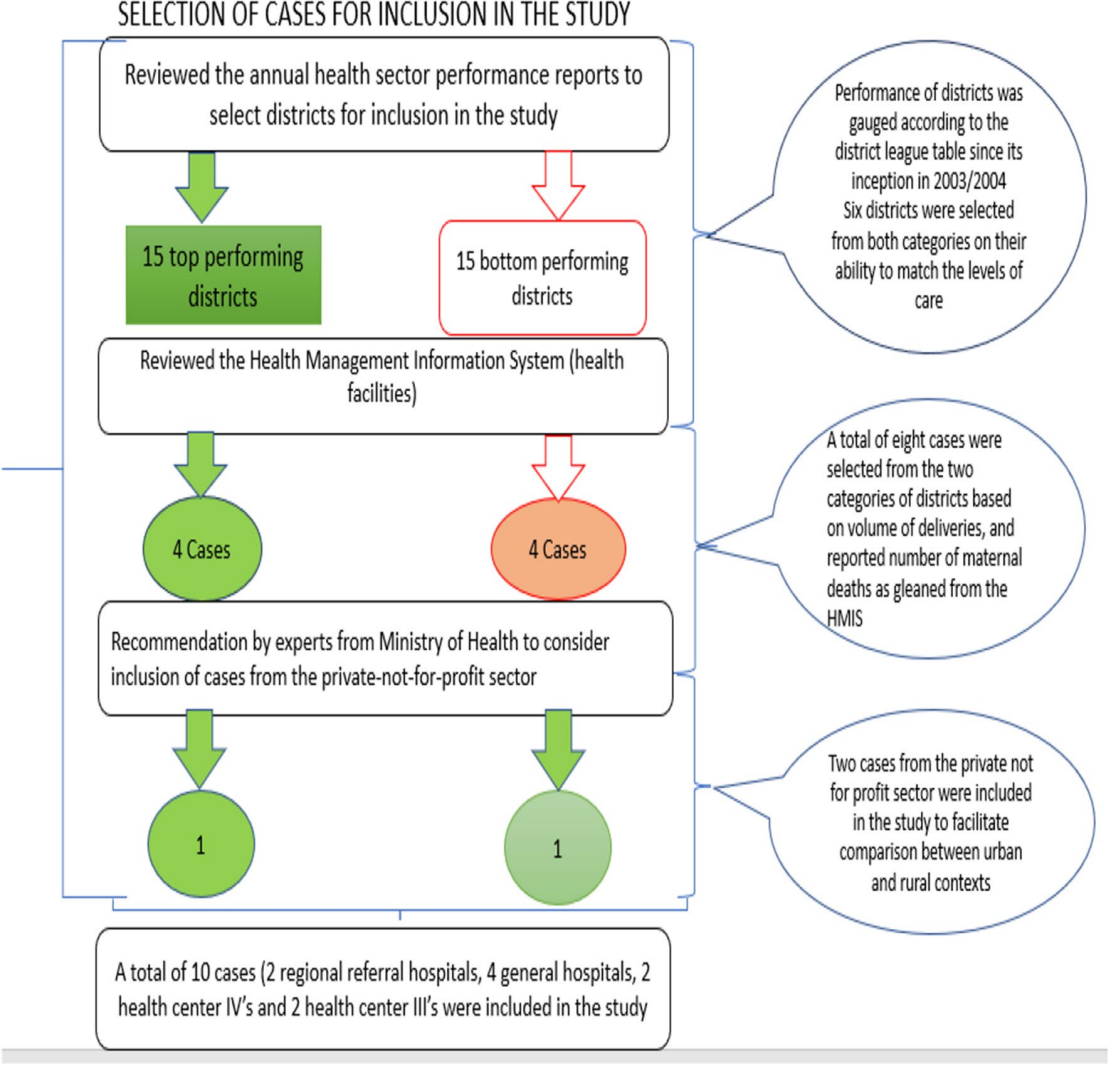
Selection of cases from top and bottom performing districts

The selected districts consistently appeared among the 15 top and 15 bottom ranked local governments (performers) on the district league table. Matching was based on the level of care and volume of maternal deliveries, as well ownership of the health facilities. For example, a district with a regional referral hospital and ranked among the 15 top performing districts on the district league table was matched against one with a regional referral hospital and also consistently appearing among 15 bottom performers at least three or more times. This same criterion was followed while selecting districts and cases representing other levels of care including general hospitals and health centre IVs and IIIs (refer to Fig. 2).

A total of six districts were initially selected for inclusion in the study. Out of these, three districts consistently appeared among the 15 top-performing districts between 2003 and 2004 and 2017 and 2018, while consistently appeared among the 15-bottom performing districts according to the district league table. An initial six cases (health facilities) were purposively selected from across these six districts. At the recommendation of the technical experts in charge of implementing the MPDSR policy at the Ministry of Health, an additional two cases were included in the study. These cases were selected from two districts that ranked among the top performing districts as per the district league table. The specific cases, however, represented the private-not-for-profit sector. According to discussions with the technical experts, they opined that the inclusion of cases from the private-not-for-profit sector would enrich the study and would facilitate comparison with the initially selected cases, which were all government-funded health facilities. Since the two private-not-for-profit cases were based in comparatively varying settings, that is, urban (capital city) and rural settings, the decision to include both in the study was also aimed at facilitating comparison to understand whether social–contextual factors, such as location, contributed to the variations in the implementation of the MPDSR policy. Also, important to note is that the selection of two cases (health centre IIIs) was done from within two districts where two general hospitals had previously been selected. This decision was informed by time and financial constraints that could not allow selection and eventual travel to another pair of districts.

Overall, 10 cases were selected on the basis of their representativeness of the various levels of care mandated by the Ministry of Health to implement the maternal and perinatal death review policy in Uganda. These include regional referral and general hospitals, health centre IVs and health centre IIIs [66]. Cases were also selected on the basis of the volume of maternal deliveries conducted at each health facility. Health facilities with the highest volume of maternal deliveries per district were included in the study. This was based on the assumption that those with the highest volumes were more likely to have higher maternal and or perinatal deaths thus making them suitable case(s) for the exploration of the study questions. The volume of maternal deliveries was ascertained from a review of the district health information system (DHIS2) maintained at the Ministry of Health resource centre.

### Selection of study participants

Study participants were purposively [74] selected from across the districts and health facilities (cases), the Ministry of Health and from agencies and professional associations including the WHO, United Nations Population Fund (UNFPA), Association of Obstetricians and Gynecologists of Uganda (AOGU), United States Agency for International Development (USAID) and the Uganda Health Service Commission (UHSC). The sampling was also informed by a review of existing documentation including the MPDSR policy guidelines as well as input from the technical experts in the field. All study participants were involved or should have been involved in the implementation of the MPDSR policy [44]. They were associated with a maternal and child health unit, department or national MPDSR committee and had worked in their current position for a period of not less than 6 months. This enabled them to suggest recommendations on strategies for addressing the causes of variations in the determinants of the implementation of the MPDSR policy within their respective health facilities. Potential study respondents who were not in position to discuss MPDSR policy or its implementation because of conflict of interest or due to confidentiality agreements were excluded from the study.

### Data collection procedures

In total, 48 people were interviewed, with the majority held at the respondents’ places of work (*n* = 45). The in-depth interviews lasted between 30 min and 2 h. An informal approach was adopted during the conduct of the interviews, which allowed the study participants to describe freely while directing the course of the discussion to broader areas that may not have been considered as useful to enriching the exploration of the study objectives [88]. Interview guides (available on request) informed by the NPT constructs were used to guide the conduct of interviews with the various categories of study participants (frontline health workers, administrative staff, representatives of agencies and professional associations and Ministry of Health staff) [49, 53]. The language was adjusted where necessary and probes were used at the discretion of the interviewer. After the first two interviews, the guide was adjusted to increase clarity and conform with maternal and child policy and clinical jargon. With permission, interviews with study participants were digitally recorded.

### Data analysis

Interviews were transcribed, deidentified and archived in NVivo data management software version 12. Each transcript was read and re-read to develop a codebook (refer to Table 1) informed by the NPT constructs and subconstructs [49, 53]. The coding process involved recognising important moments and encoding them to organise data and identify themes and patterns [12]. D.R.W. then undertook an exploration of how the emerging themes fitted within the four major NPT constructs and their respective subconstructs using NVivo. Placing the emerging themes under the NPT constructs enabled using the theory to shape the potential interpretations of the research findings, followed by compiling analytical notes on the observed similarities and differences in the determinants of implementation of the MPDSR policy.

**Table 1:** Codebook informed by NPT constructs and subconstructs

Name of code (parent node/construct)	Name of the subcode (child node/subconstruct)	Explanation of the codes
Coherence	The sense-making work that people do individually and collectively to operationalise the implementation of MPDSR (sense-making efforts)	
	Differentiation	How implementing the MPDSR policy is different from other interventions aimed at improving maternal and child health
	Communal specification	How people working together build a shared understanding of the aims, objectives, and benefits of implementing MPDSR
	Individual specification	How participants collaboratively need to do things that will help them understand their specific tasks and responsibilities around the implementation of MPDSR policy
	Internalisation	How participants in sense making undertake efforts to understand the value, benefits, and importance of implementing the MPDSR policy
Cognitive participation	How people engage and participate in the implementation of MPDSR policy (relational efforts)	
	Initiation	Whether or not the key participants are driving forward the implementation of MPDSR
	Enrolment	How participants organise and reorganise themselves to collectively contribute to the work involved in implementing the MPDSR policy
	Legitimation	Ensuring that other participants believe it is right for them to be involved and that they can make a valid contribution
	Activation	Actions and procedures needed to sustain implementation of MPDSR policy as collectively defined by the participants
Collective action		Operational work that people do to enact MPDSR policy (operational efforts)
	Interactional workability	Interactional work that people do with each other and with elements of implementation of MPDSR when they seek to operationalise it in everyday settings
	Relational integration	Knowledge work that people do to build accountability and maintain confidence in the implementation of MPDSR and in each other as they implement the policy
	Skillset workability	Allocation of work that underpins the division of labour that is built up around implementation of MPDSR as it is operationalised in the real world
	Contextual integration	Resource work – managing implementation of MPDSR through the allocation of different kinds of resources and execution of protocols, policies and procedures
Reflexive monitoring	Appraisal work that people do to assess and understand the ways that MPDSR policy implementation affects them and those around them (appraisal efforts)	
	Systematisation	Determining the usefulness of implementing MPDSR for the participants and for the others and involves the work of collecting information in a variety of ways
	Communal appraisal	Working together – sometimes in informal collaboratives, sometimes in informal groups to evaluate the worth of a set of practices
	Individual appraisal	How individuals involved in the implementation of MPDSR work experientially as individuals to appraise its effects on them and the contexts in which they are set
	Reconfiguration	Whether appraisal work by individuals or groups leads to attempts to redefine procedures or modify practices and to event to change/update the MPDSR guidelines and policy

## Results and discussion

**Fig. 3:**
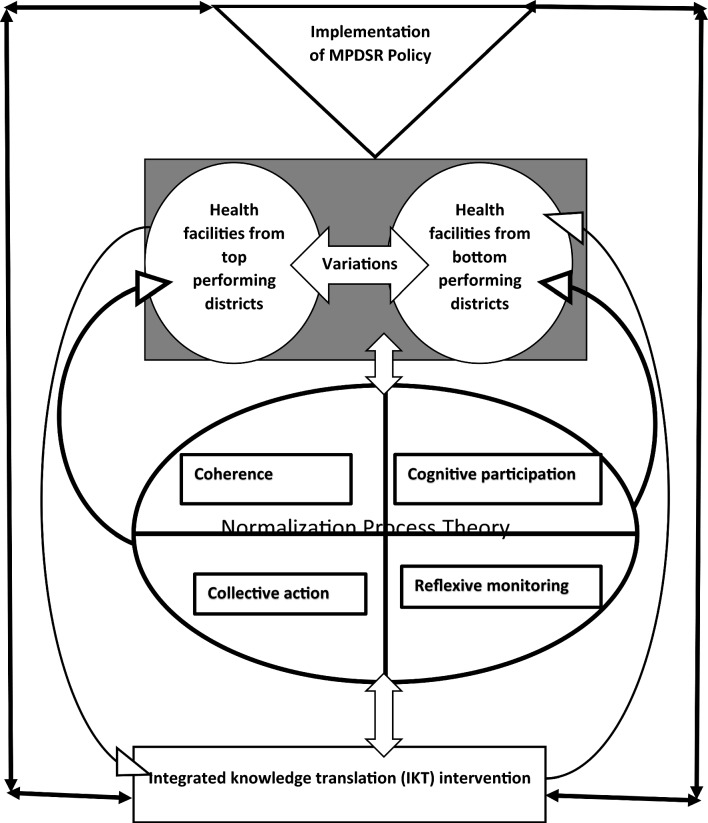
Exploring variations in the implementation of a health systems level policy intervention to improve maternal and child health: a conceptual framework

Descriptive characteristics of the 48 participants and how their actions accounted for the observed variations in the implementation of the MPDSR policy are reported in detail elsewhere [91]. Figure 3 provides a schematic illustration of how NPT was used to explore the variations in the implementation of the MPDSR Policy in Uganda.

As a national level policy intervention, the MPDSR policy is meant to be implemented by all health centre IIIs, IVs, general hospitals, regional referral hospitals and national regional referral hospitals across the country’s health system (represented by the greyscale background) (refer to Fig. 3) [65]. As recommended by the policy, some health facilities from mostly top performing districts have consistently notified the Ministry of Health regarding facility related maternal and perinatal deaths (MPDs) [63]. These were categorised as consistent health facilities. However, there were also another category of health facilities mostly from bottom performing districts that were struggling with notifying the Ministry of Health regarding MPDs as recommended by the MPDSR policy [63]. These were categorised as inconsistent health facilities. This study explored the variations in the implementation of the MPDSR policy between health facilities selected from top performing and bottom performing districts using the NPT.

### How was NPT used to explore the implementation of the MPDSR policy?

NPT constructs and subconstructs were applied to understand the sense-making, cognitive participation, collective action and reflexive monitoring efforts that participants across selected facilities at the different levels of care involved in implementing the MPDSR policy. Specifically, NPT was utilised in several ways including informing the study design, (generation of the specific research questions), structuring the data collection tools (semi-structured interview guides), providing an organising framework for analysis, interpreting and reporting of study findings, as well as making recommendations. Additionally, the theory facilitated identification factors that may help to explain the variations in the implementation of the policy. Despite these contributions, there were also noted limitations as described in detail below.

### Informing the study design, data collection and analysis

NPT constructs and subconstructs were used to provide conceptual vocabulary that facilitated exploration of the variations in the determinants of implementation of the MPDR policy [52]. For example, with regard to the generation of specific research questions, those relating to how actors/stakeholders involved in the implementation of the policy made sense of its implementation including differentiating its implementation from other interventions, the efforts they invested in building a community of practice around the implementation of the policy, their collective and appraisal efforts were informed by the various NPT constructs and subconstructs. This resonates with previous studies that have reportedly used NPT to generate research questions for fieldwork [27, 32, 78]. Additionally, coupled with the previously reported correlations between and among the constructs [18, 90], the underlying assumptions about how the constructs could facilitate exploration of the study questions influenced the design and structure of the interview guides. For example, it was assumed that getting a glimpse into how participants made sense of the intervention would enable exploration of how they organise themselves to drive it forward, their collective as well as appraisal efforts. As such questions were structured to facilitate exploration of the entire process-from sense making, cognitive participation, collective action and appraisal efforts. However, the actual interviews generally followed their own course; although, the interview guides always helped to keep the discussion on track. Furthermore, probes used throughout the interview guide mostly benefited from the language used by the authors and previous users of NPT.

Relatedly, NPT constructs and subconstructs provided an organising framework for analysis, interpretation and reporting of study findings as well as making recommendations, especially the deductive coding and analysis of data, as they were used as parent and child nodes in NVivo. Upon analysis, study findings were reported on the basis of how participants invested efforts across the four major NPT constructs. This approach compares to that by McEvoy et al. [57] who observed that in almost all the 29 studies included in their systematic review, NPT was used as an organising framework for analysis, reporting of findings and informing study intervention design [24, 34, 45, 57]. Additionally, while making recommendations, participants were requested to highlight what can be done to enhance the sense making, cognitive participation, collective and appraisal efforts invested in the implementation of the MPDSR policy. Detailed recommendations under each construct are reported elsewhere [91].

### Identification of factors to explain variations in implementation of MPDSR

NPT was used to identify factors that may explain the observed variations in the implementation of the MPDSR policy across various social settings. As such, the theory went beyond the description of barriers and facilitators and enabled exploration of how the efforts that actors involved in the implementation of the MPDSR policy affect its embedding within the various social contexts [49]. For example, by facilitating exploration of how the differences in the levels of the sense-making, relational, operational and appraisal efforts invested by actors across the different levels of care and between top and bottom performing districts might explain the observed variations in the implementation of the policy, the theory provided valuable insights regarding the need to invest efforts across the four NPT constructs [18, 90]. Findings from this study further demonstrated that though conceptually distinct, the NPT constructs are interconnected, can help to depict elements of the implementation processes and can be applied in a stable and consistent way to explore variations within and between cases [52].

### Exploration of contextual factors

Despite criticisms levied against NPT, such as over emphasis on individual and collective agency at the expense of context [15, 80], findings from this study further revealed that the theory facilitated exploration of contextual factors such as hard-to-reach areas, lack of access to affordable health services, neighbourhood poverty and lack of access to education, which can potentially explain the observed variations in the implementation of the policy [91]. Although Nilsen [71] attributes the limited articulation of how such contextual factors affect implementation of interventions to the complexities in understanding of context as a moderator of change in health care organisations as well as the lack of a unifying definition for context in implementation science and related fields [71], findings from this study successfully illustrate the utility of NPT in exploring such factors.

### Limitations of NPT

Amidst the reported utility of NPT in facilitating exploration of the variations in the implementation of the MPDSR policy, there were also observed limitations, some of which have also been alluded to by previous studies. Among these are the existence of overlaps across different NPT constructs and subconstructs [1, 30, 43, 45]; the limited utilisation of the theory in prospective studies [52, 57], as well as the inability of the theory to explore relationships between individual attitudes, intentions and outcomes which is a concern of psychological theories such as Theory of Planned Behaviour Change [2, 57]. Within the confines of this study, the observed limitations such as the overlaps between constructs and subconstructs complicated coding and analysis of variables that were crosscutting in nature. Additionally, efforts to prospectively explore the implementation of the policy among cases that were not yet implementing the policy (despite being mandated by MoH) were constrained as differentiating an intervention from similar interventions requires initial exposure. Therefore, without prior exposure to the MPDSR policy, participants from such cases were challenged at articulating the sense-making, relational, operational and appraisal efforts.

## Conclusions

By informing the study design, development of data collection tools, analysis, interpretation and reporting of findings, NPT enabled identification of the factors that facilitated exploration of the variations in the implementation of the MPDSR policy across various social settings. Findings from this study demonstrate the suitability and utility of the theory in exploring implementation of health system policy interventions. However, while NPT sufficiently guided exploration of the implementation of the MPDSR policy, it mostly focuses on the agency of those involved in implementation at the expense of the recipients/beneficiaries of the intervention [52, 80]. As such, to develop the theory further, efforts are required to understand how those who experience the effects of the agency (recipients of intervention) influence whether the intervention becomes embedded within the various contexts or not.

## Data Availability

The datasets generated and/or analysed during the current study are not publicly available because it was a qualitative study but are available from the corresponding author on reasonable request.

## Abbreviations

AOGU: Association of Obstetricians and Gynaecologists of Uganda
DHMIS: District Health Management Information System
DHO: District Health Officer
HC: Health Center
HDREC: Higher Degrees, Research and Ethics Committee
HMIS: Health Management Information System
HSD: Health subdistrict
IDI: In-depth interview
IMR: Infant mortality rate
LMICs: Low- and middle-income countries
MDSR: Maternal Death Surveillance and Response
MDGs: Millenium Development Goals
MMR: Maternal mortality ratio
MNCAH: Maternal newborn child and adolescent health
MNH: Maternal and newborn health
MOH: Ministry of Health
MoLG: Ministry of Local Government
MDR: Maternal death reviews
MPDR: Maternal perinatal death reviews
MPDSR: Maternal and Perinatal Death Surveillance and Response
NPT: Normalisation process theory
NRH: National referral hospitals
PDR: Perinatal death reviews
PHP: Private health practitioners
PNFP: Private not for profit
REC: Research ethics board
RRHs: Regional Referral Hospitals
SDGs: Sustainable Development Goals
TCMPs: Traditional complementary medicine practitioners
UHSC: Uganda Health Service Commission
UNCST: Uganda National Council for Science and Technology
UNHS: Uganda National Health System
UNFPA: United Nations Populations Fund
USAID: United States Agency for International Development
WHO: World Health Organization
